# A Case of Suppression of Salivary Secretion

**Published:** 1877-10

**Authors:** Donald Baynes


### A CASE OF SUPPRESSION OF SALIVARY SECRETION.

BY DR. DONALD BAYNES.

(*Translated by S.*)

In the month of last November, I had occasion to observe a curious case of suppression of salivary secretion.

The subject of this observation, J. B., aged twenty-two years, was taking care of horses at an inn. He presented himself to me November 10th, 1876. He complained of great dryness of the mouth, of an impossibility to swallow food, unless he took great quantities of liquid to carry away the aliment.

The patient made vain efforts to expectorate. He complained of his tongue feeling swollen, and his mouth filling up with thickened mucous. He had been obliged to wash his mouth, and to drink night and day. He had not been able to sleep more than an hour at a time, but was obliged to rise and rinse his mouth. He also complained of a suffocating sensation.

On examining the mouth, I found that the tongue was swollen, and covered with a thick, whitish coating. A mass of matter which reminded me of the soft roe of a fish. Offensive matter was massed between the lips and covered the jaw.

I feared at first a fungous growth, as well as parasitic, but upon examining with the microscope, saw that it was amylaceous granulations. The coating was simply composed of vitiated food which accumulated at this place, and was not raised up in consequence of want of secretion of saliva.

The duct or canal of Steno and of Wharton, remained open, and no obstruction seemed to be able to explain the want cf secretion.

The general health was excellent. However, the 20th of October the patient had taken cold, and had tonsil quinsy, which yielded to ordinary treatment.

The suppression of saliva lasted more than six months, during which I employed, without effect, some stimulating gargles, nux vomica, iodide of potash, aperients, etc.

The 28th of November I introduced into the duct of Steno a metallic probe, which I put in contact with the negative pole of a battery, and applied the positive pole to the nape of the neck.

During the application of electricity, which lasted ten minutes, I frequently reversed the current.

It was not more than three minutes after the application, the patient cried out, "My mouth is moist."

He returned the following day and said he had slept all night, and felt that his mouth was much better. I renewed the electricity, which produced an abundance of saliva.

Since this time the patient has had no return of the strange symptoms, for which he came to consult me.

*Gazette des Hopitaux.*

